# Mortalidade e Arritmia Ventricular em Pacientes com Repolarização Precoce

**DOI:** 10.36660/abc.20240516

**Published:** 2025-04-17

**Authors:** Hugo Baldisserotto, Barbara Adelmann de Lima, Marco Aurélio Lumertz Saffi, Anderson Donelli da Silveira, Marcia Leonardi Baldisserotto, Tiago Luiz Luz Leiria

**Affiliations:** 1 Programa de Pós-Graduação em Ciências da Saúde: Cardiologia e Ciências Cardiovasculares Universidade Federal do Rio Grande do Sul Porto Alegre RS Brasil Programa de Pós-Graduação em Ciências da Saúde: Cardiologia e Ciências Cardiovasculares, Universidade Federal do Rio Grande do Sul, Porto Alegre, RS – Brasil; 2 Pontifícia Universidade Católica do Rio Grande do Sul Porto Alegre RS Brasil Pontifícia Universidade Católica do Rio Grande do Sul, Porto Alegre, RS – Brasil; 3 Departamento de Psicometria Universidade Federal do Rio de Janeiro Rio de Janeiro RJ Brasil Departamento de Psicometria, Universidade Federal do Rio de Janeiro (UFRJ), Rio de Janeiro, RJ – Brasil

**Keywords:** Eletrocardiografia, Mortalidade, Arritmias Cardíacas

## Abstract

**Fundamentos:**

Estudos recentes associaram a repolarização precoce (RP) com um risco de fibrilação ventricular aumentado, especialmente nas derivações V1-V3 e nas regiões inferior e lateral. No entanto, dados sobre a população brasileira são limitados.

**Objetivos:**

Estimar o impacto da RP na sobrevida e na ocorrência de arritmias ventriculares (AVs) em um período de 10 anos em um hospital universitário.

**Métodos:**

Este estudo retrospectivo do tipo coorte incluiu pacientes com RP no eletrocardiograma a partir do banco de dados do hospital. Análise descritiva foi conduzida para descrever o perfil e as características dos pacientes. O método de Kaplan-Meier foi usado para avaliar as diferenças entre os tipos de RP. Modelos de regressão de Cox foram aplicados para avaliar os riscos de morte e AV. calculando os hazard ratios brutos e ajustados. O nível de significância adotado na análise estatística foi de 5%.

**Resultados:**

A população do estudo foi predominantemente do sexo masculino, com idade média de 45,6 anos; 2,7% apresentaram AV (cinco no grupo com RP lateral, quatro no grupo com RP inferior e quatro no grupo com RP ínfero-lateral). Foram observadas diferenças significativas na idade, sexo, e ocorrência de insuficiência cardíaca com fração de ejeção reduzida entre os grupos. Em relação à mortalidade por todas as causas, 2,3% dos pacientes foram a óbito (cinco no grupo com RP lateral, um no grupo com RP inferior e cinco no grupo com RP ínfero-lateral). Somente idade mostrou uma diferença estatisticamente significativa. Observou-se diferenças significativas na ocorrência de morte e de AV entre os grupos (p=0,7 e p=0,5, respectivamente).

**Conclusão:**

A RP não levou a uma maior incidência de AV nem de mortalidade por todas as causas na população estudada.

## Introdução

A prevalência de repolarização precoce (RP) varia entre 2,3% e 29,3%, variando conforme idade, raça e sexo.^1^ A RP é comumente observada em atletas de alto desempenho, com uma incidência de cerca de 1/3. No entanto, a incidência anual de morte cardíaca súbita nessa população é muito baixa.^2^ Por muitos anos, a RP foi considerada um achado benigno no Eletrocardiograma (ECG); porém, estudos recentes sugeriram que esse padrão em derivações diferentes de V1-V3 (principalmente derivações inferiores) está associado com um risco maior de arritmias ventriculares e morte súbita.^1,3-5^

Haïssaguerre et al.^4^encontraram uma alta prevalência de RP em pacientes que foram ressuscitados após parada cardíaca causada por fibrilação ventricular (FV) abortada, e os pacientes com RP apresentaram um risco duas vezes maior de recorrência de FV.^4^ No estudo CASPER, os pacientes que apresentaram morte súbita abortada e coração estruturalmente normal, a RP foi a causa em 7,9% da amostra.^6^ O grau de elevação do ponto J nas derivações inferiores pode se correlacionar om a magnitude do risco. O risco relativo de morte foi 1,3 naqueles com 0,1mV e três vezes naqueles com 0,2mV;^1^ o risco de arritmia ventricular foi maior nos pacientes com 0,2 mV, com a inclinação do segmento ST resultando em um prognóstico significativo, com uma orientação horizontal ou descendente associada com um risco maior de arritmia.^7^

O rastreamento e o manejo dos pacientes com RP e o risco de morte súbita são um desafio.^8^ A *American Heart Association* (AHA) recomenda que, se detectada, a RP deve ser investigada em caso de síncope não explicada ou história familiar de morte súbita e características de ECG arritmogênico.^8^ A baixa incidência de eventos arrítmicos é também um desafio importante para intervenções efetivas que pode reduzir o risco de arritmia.^2^

Embora quinidina e isoproterenol possam ser efetivas no manejo de pacientes com FV, os dados que apoiam seu uso continuam limitados a amostras pequenas.^9,10^ Devido aos dados limitados na literatura, principalmente em relação à população brasileira, este estudo tem como objetivo avaliar se pacientes com diferentes tipos de RP têm um risco maior de arritmias ventriculares e mortes.

## Métodos

Mudanças temporais nos padrões de RP não foram avaliadas neste estudo; somente um ECG foi avaliado. A RP foi classificada com base na morfologia observada nas derivações: lateral (derivações I, aVL, V5-V6), inferior (derivações II, III, aVF), e ínfero-lateral (combinação dessas derivações).

Este estudo retrospectivo do tipo coorte incluiu 478 pacientes de um hospital universitário com um diagnóstico eletrocardiográfico de RP precoce entre 2011 e 2022. O estudo foi conduzido de acordo com diretrizes regulatórias envolvendo seres humanos, e o projeto de pesquisa foi aprovado pelo Comitê de Ética em Pesquisa institucional (número 2021/0585).

**Figura 1:**
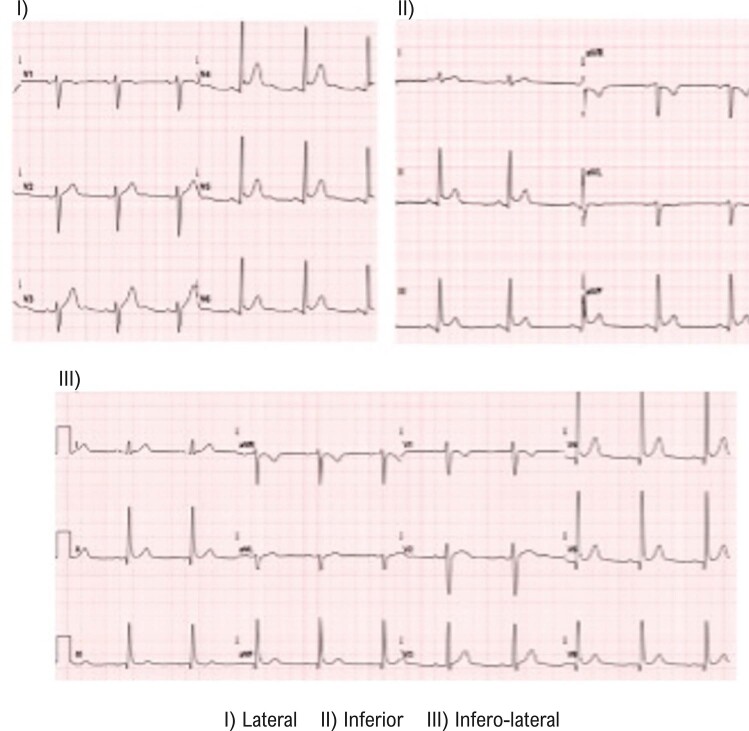
– Padrões eletrocardiográficos de repolarização precoce.

Os critérios de inclusão foram pacientes diagnosticados (por cardiologistas) com repolarização ventricular precoce – elevação do ponto J ≥ 1 mm em duas ou mais derivações consecutivas, com um padrão de depressão (*slurring*) ou de entalhe (*notching*), excluindo as derivações V1-V3 (Figura 1), submetidos a um ECG no hospital universitário e com idade superior a 18 anos entre 2011 e 2022. Os critérios de exclusão foram impossibilidade de se realizar um ECG para medir os parâmetros de repolarização ventricular, alterações secundárias da repolarização ventricular, e pacientes com marcapassos e outros dispositivos cardíacos.

A busca pelo termo “repolarização precoce” no banco de dados do hospital resultou em 2172 pacientes. Desses, 923 foram selecionados para revisão com base nos critérios de inclusão. Após avaliação completa, 478 pacientes foram diagnosticados com RP.

### Variáveis do estudo

As variáveis de exposição incluíram repolarização ventricular precoce, classificada em três grupos: lateral, inferior e ínfero-lateral. Variáveis de desfecho incluíram arritmia ventricular sustentada e mortalidade por todas as causas. Variáveis de confusão controladas foram idade e sexo.

### Análise dos dados

Foi realizada uma análise exploratória das características da amostra, e os resultados apresentados em proporções ou médias, dependendo da natureza da variável. Subsequentemente, as características da amostra foram estratificadas de acordo com mortes e arritmias ventriculares para análise posterior. Diferenças entre os grupos foram avaliadas pelo teste do qui-quadrado para dados categóricos e pelo teste t para dados contínuos, com significância estatística estabelecida em p ≤ 0,05. Uma população controle foi usada para análise comparativa.

Para a análise de sobrevida considerando os vários tipos de RP, o tempo foi calculado em meses (0-120 meses), e o evento (falha) foi definido como óbito do paciente durante o seguimento de 10 anos. Os pacientes que sobreviveram além do período de acompanhamento foram censurados à direita. Para a análise do tempo até a ocorrência de arritmia ventricular, o tempo foi calculado em meses, e o evento (falha) foi definido como ocorrência de arritmia durante o seguimento de 10 anos. Os pacientes com arritmia ventricular após o período de seguimento foram considerados dados censurados à direita.

Para estimar a probabilidade de sobrevida e o risco cumulativo ao longo do tempo para morte e arritmia ventricular em relação aos tipos de RP, foi empregado o método de Kaplan-Meier. Diferenças entre curvas de sobrevida para ambos os desfechos foram avaliadas usando o teste de log-rank, com significância estabelecida em p≤0,05.

Para analisar a relação entre RP e o tempo até ocorrência de morte ou arritmia na população do estudo, foi usada a regressão de Cox. *Hazard ratios* (HRs) brutos e ajustados foram obtidos para as variáveis de confusão, além do Intervalo de Confiança de 95% (IC95%). A análise dos dados foi realizada usando o programa R versão 4.3.0.

## Resultados

Durante o acompanhamento, as arritmias ventriculares incluíram taquicardia ventricular sustentada e fibrilação ventricular conforme definições da CID-10. As causas de óbito foram condições cardíacas primárias, incluindo parada cardíaca súbita.

**Figura 2:**
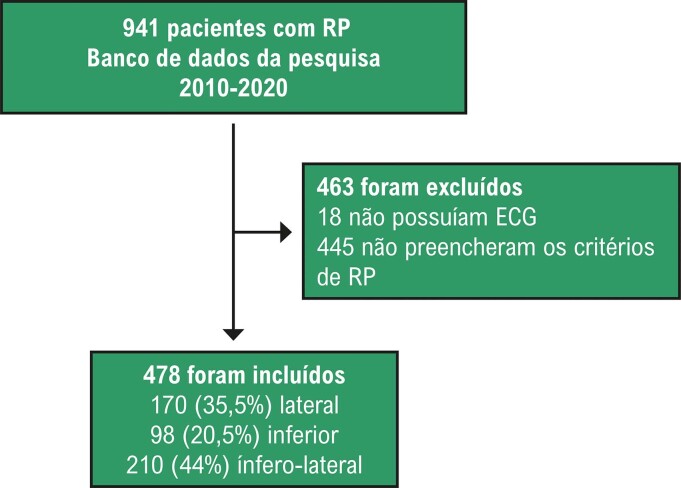
– Fluxograma da seleção dos pacientes; ECG: eletrocardiograma; RP: repolarização precoce.

Um total de 2172 pacientes com diagnóstico prévio de RP no ECG foram inicialmente identificados do banco de dados do hospital. Desses, 941 foram selecionados para revisão com base nos critérios de inclusão. Após avaliação por três cardiologistas, 478 pacientes foram confirmados com diagnóstico genuíno de RP, e foram incluídos no estudo. Os outros pacientes que não preencheram os critérios de RP foram usados como controle (Figura 2 e Figura Central).

**Figura Central:**
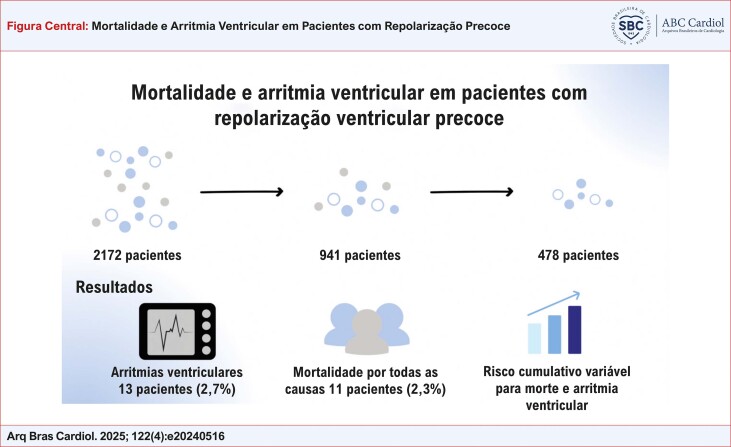
: Mortalidade e Arritmia Ventricular em Pacientes com Repolarização Precoce

A população do estudo era predominantemente do sexo masculino (745), com uma idade média de 45,6 anos. Entre eles, 21,5% eram tabagistas, e 43% tinham ensino médio incompleto. A prevalência de comorbidades tais como diabetes mellitus, hipertensão arterial sistêmica, dislipidemia, doença arterial coronariana, e insuficiência cardíaca com fração de ejeção reduzida variou na amostra, com o grupo com RP lateral mostrando uma prevalência mais alta de hipertensão arterial sistêmica (Tabela 1).

**Tabela 1:** – Características da população do estudo

	Lateral	Inferior	Ínfero-lateral	Total
**Variáveis**	(n = 170)	(n = 98)	(n = 210)	n = 478
**Idade**	48,5±4,95	45,5±7,78	43,2±5,46	45.57±6.56
**Escolaridade**				
Fundamental incompleto	79 (46)	40 (41)	87 (41)	206
Fundamental completo	36 (20,4)	19 (19)	45 (21)	100
Ensino médio completo	29 (17)	19 (19)	38 (18)	86
Ensino superior completo	11 (6,4)	9 (9,1)	12 (5,7)	32
Desconhecido	7 (4,1)	9 (9,1)	17 (8,9)	33
**Gênero**				
Masculino	130 (76)	70 (71)	154 (73)	354
Feminino	40 (24)	28 (29)	56 (27)	124
**Diabetes Mellitus**	35 (20)	16 (16)	42 (20)	93
**HAS**	86 (50)	32 (33)	75 (36)	193
**Dislipidemia**	14 (8,2)	5 (5,1)	12 (5,7)	31
**Tabagista**	35 (20)	19 (19)	49 (23)	103
**DAC**	17 (10)	12 (12)	14 (6,7)	43
**ICFEr**	5 (3)	2 (2)	2 (1)	9

HAS: hipertensão arterial sistêmica; DAC: doença arterial coronariana; ICFEr: insuficiência cardíaca com fração de ejeção reduzida.

Durante o período de acompanhamento, 13 pacientes (2,7%) apresentaram arritmias ventriculares, com incidência variável entre os grupos com RP. Foram observadas diferenças significativas em idade, sexo, e insuficiência cardíaca com fração de ejeção reduzida entre os grupos. Quanto à mortalidade, 11 pacientes (2,3%) foram a óbito, com diferenças de idade entre os grupos com RP (Tabela 2).

**Tabela 2:** – Características dos pacientes (n=11) que foram a óbito durante o acompanhamento

	Morte por todas as causas		Arritmia ventricular	
	(n=11)	Valor p^**c**^	(n=13)	Valor p^**d**^
**Idade**	59±10,24	0,0012^b^	35,3±17,50	0,0519^b^
**Escolaridade**		0,3140^a^		0,2813^a^
Fundamental incompleto	3 (27)		8 (62)	
Fundamental completo	2 (18)		2 (15)	
Ensino médio completo	6 (55)		3 (23)	
Ensino superior completo	0		1 (8)	
Desconhecido	0		0	
**Tipo de RP**		0,6201^a^		0,4758^a^
Lateral	5 (45)		5 (38)	
Inferior	1 (9)		4 (31)	
Ínfero-lateral	5 (45)		4 (31)	
**Sexo**		1^a^		0,0255^a^
Masculino	8 (73)		13 (100)	
Feminino	3 (27)		0	
**Diabetes mellitus**	2 (18)	1^a^	1 (8)	0,4786^a^
HAS*	5 (45)	0,9709^a^	6 (46)	0,7765^a^
**Dislipidemia**	0	1^a^	1 (8)	0,5865^a^
**Tabagista**	3 (27)	0,8334^a^	2 (15)	0,1588^a^
**DAC**	0	0,6099^a^	3 (23)	0,1031^a^
**ICFEr**	1 (9)	0,4504^**a**^	2 (15)	0,0221^a^

*HAS: hipertensão arterial sistêmica; DAC: doença arterial coronariana; ICFEr: insuficiência cardíaca com fração de ejeção reduzida; RP: repolarização precoce; em negrito valores de p < 0,05. ^a^: teste do qui-quadrado; ^b^: teste t de student ; ^c^: em comparação ao grupo de pacientes que não foram a óbito ; ^d^: em comparação ao grupo de pacientes sem arritmia ventricular.

**Figura 3:**
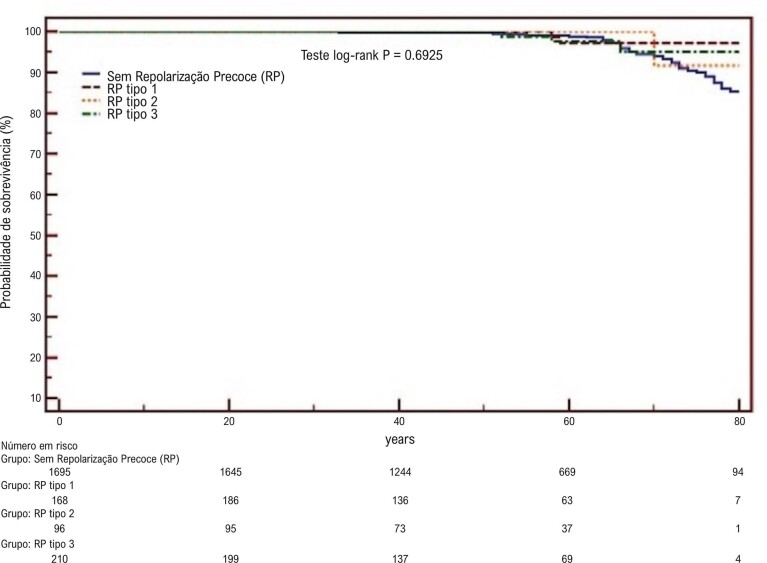
– Curvas de sobrevida de Kaplan-Meier; RP: repolarização precoce.

A análise de sobrevida de Kaplan-Meier demonstrou não haver diferença estatisticamente significativa nas taxas de sobrevida entre os grupos de pacientes com diferentes tipos de RP e o grupo controle (valor p = 0,7 e valor p = 0,5 para mortalidade e arritmia ventricular, respectivamente). No entanto, a análise revelou risco cumulativo variável para morte e arritmia entre os diferentes grupos de RP (Figura 3).

A análise de regressão de Cox mostrou ausência de razões de risco (*hazard ratios*) estatisticamente significativos para os diferentes tipos de RP quanto à mortalidade ou arritmias ventriculares. Somente idade demonstrou uma razão de risco significativa para ambos os desfechos (Tabela 3).

**Tabela 3:** – Modelos brutos e ajustados (regressão de Cox) da relação da repolarização precoce com morte e arritmia ventricular da população estudada

	Morte		Arritmia	
	HR Bruto	HR Ajustado	HR Bruto	HR Ajustado
**Repolarização precoce**				
Lateral	HR= 1,000	HR=1,000	HR=1,000	HR= 1,000
Inferior	HR=0,419 (IC= 0,049 - 3,589)	HR= 0,528 (IC= 0,061 - 4,580)	HR= 1,537 (IC= 0,412 - 5,731)	HR= 1.278 (IC= 0,336 - 4,856)
Ínfero-lateral	HR=0,928 (IC= 0,268 - 3,211)	HR= 1,242 (IC= 0,354 - 4,365)	HR= 0,670 (IC= 0,180 - 2,497)	HR= 0,543 (IC= 0,143 - 2,054)
**Idade**		HR= 1,080 (IC= 1,027- 1,135)	HR= 0,959 (IC= 0,925 - 0,995)	
**Sexo**				
Masculino		HR=1,000		
Feminino		HR= 0,988 (IC= 0,2605 - 3,750)		

## Discussão

Achados recentes de Liu et al.^11^ sugerem que a RP seja um fenômeno transiente, aparecendo em ECGs basais, mas potencialmente se resolvendo em medidas subsequentes. Tal natureza transiente poderia indicar um curso benigno, conforme discutido em seu estudo coorte.

A elevação do ponto J no ECG nas 12 derivações, compatível com RP, tem sido tradicionalmente vista como um achado inocente em indivíduos sadios.^12,13^

A amostra do estudo, como esperado, consistiu em indivíduos de meia idade com um número menor de comorbidades em comparação aos tipicamente relatados em estudos de cardiologia. Embora não seja um estudo randomizado, as características dos grupos foram muito similares, e somente o grupo com RP lateral mostrou uma incidência mais alta de paciente hipertensos, reduzindo o efeito de viés de seleção, embora ainda presente. Estudos prévios relataram idades médias de populações com RP igual ou muito similar à da amostra deste estudo.^1^

Ao analisar as características dos pacientes que foram a óbito, a idade resultou em uma diferença estatisticamente significativa, confirmando a associação esperada entre idade mais avançada e maior risco de mortalidade. Quanto à incidência de arritmias ventriculares, sexo e Insuficiência Cardíaca com Fração de Ejeção Reduzida (ICFEr) resultaram em diferenças estatisticamente significativas. Os pacientes com arritmia ventricular consistiram em pacientes jovens, do sexo masculino, com uma notável porcentagem de pacientes com ICFEr, indicando uma predisposição à arritmogênese por meio de vários mecanismos de cardiomiopatia. Idade, após ajuste no modelo Cox, apresentou-se como um fator protetor contra arritmia ventricular.^3^

Curvas de sobrevida analisadas usando o método de Kaplan-Meier revelaram altas taxas de sobrevida em todos os grupos, mas sem diferença estatisticamente significativa (p=0,7). No entanto, o grupo com RP ínfero-lateral mostrou o risco cumulativo mais alto. Consistente com os achados do estudo de Tikkanen et al.^1^ em 2009, os pacientes com RP inferior mostraram uma taxa mais alta de mortalidade cardiovascular, principalmente quando o ponto J era maior que 2mm. No entanto, ao analisar a mortalidade por todas as causas, não foi observada diferença estatisticamente significativa entre os grupos com RP lateral e RP inferior. A porcentagem da amostra sem arritmia ventricular foi alta em todos os grupos, sem diferenças estatisticamente significativas (p=0,5). Diferentemente da mortalidade, os pacientes com RP inferior mostraram um risco cumulativo mais alto de arritmia ventricular, enquanto os pacientes om RP ínfero-lateral apresentaram o risco mais baixo. Achados similares foram relatados por Tikkanen et al.^1^ em 2009 sobre arritmia ventricular, com uma maior incidência observada no grupo com RP inferior quando o ponto J era maior que 2mm.^1^

Embora a mortalidade não tenha sido estatisticamente diferente entre os grupos, foi interessante notar a diferença observada no HR ajustado para idade e sexo no grupo com RP ínfero-lateral. Esse resultado sugere um fator de risco potencial, com uma chance 1,2 vezes maior de morte. Contudo, o amplo intervalo de confiança, incluindo o zero, pode ser atribuído ao pequeno número de mortes. Mais recentemente, Haïssaguerre et al.^4^ observaram uma frequência mais alta de RP ínfero-lateral em pacientes com morte súbita abortada por taquicardia ventricular, corroborando os achados deste estudo, que a categoria de RP está associada com um risco maior de arritmia ventricular e morte.^4^

Apesar das importantes contribuições a esta área do conhecimento, o presente estudo possui várias limitações. Os dados foram obtidos retrospectivamente por meio da análise dos prontuários médicos, e muitos detalhes dependeram das descrições de terceiros, que às vezes resultou em informações incompletas ou faltantes. A extração de dados dos prontuários médicos também foi limitada por questões técnicas e acesso restrito a esses documentos. Ainda, o uso de dados secundários, tais como o SIM, dadas as informações escassas sobre óbitos, dificulta a categorização das causas de óbito. Outra limitação foi o pequeno número de desfechos (morte e arritmia ventricular), o que pode comprometer o poder da amostra para detectar diferenças estatisticamente significativas. É essencial que médicos clínicos, particularmente cardiologistas, reconheçam a importância desta nova evidência, e se atentem quando os pacientes apresentarem esses achados eletrocardiográficos. O tamanho amostral relativamente pequeno e o número limitado de desfechos, particularmente morte e arritmias ventriculares, pode haver impactado o poder estatístico do estudo e a capacidade de detectar diferenças significativa entre os grupos com RP. Ainda, a natureza unicêntrica do estudo e os critérios de exclusão podem limitar a possibilidade de extrapolar os achados para populações mais amplas. A qualidade e a disponibilidade dos dados, bem como os potenciais fatores de confusão não completamente controlados, adicionam complexidade à interpretação dos resultados. A natureza observacional do estudo impede o estabelecimento de relações causais entre RP e os desfechos. Períodos mais longos de acompanhamento, e estudos prospectivos multicêntricos são necessários para validar os achados e abordar essas limitações.

## Conclusões

No presente estudo, observamos que os pacientes com repolarização ventricular precoce, particularmente nas derivações inferiores e laterais, não apresentaram uma incidência significativamente mais alta de arritmias ventriculares ou mortalidade. Esses resultados sugerem que a presença isolada de RP pode não ser um forte preditor de desfechos adversos na população de nosso estudo. Contudo, outros estudos prospectivos e randomizados são necessários para validar esses resultados e direcionar a prática clínica quanto ao manejo dos pacientes com repolarização ventricular precoce.

## References

[1] Tikkanen JT, Anttonen O, Junttila MJ, Aro AL, Kerola T, Rissanen HA (2009). Long-Term Outcome Associated with Early Repolarization on Electrocardiography. N Engl J Med.

[2] Maron BJ (2003). Sudden Death in Young Athletes. N Engl J Med.

[3] Klatsky AL, Oehm R, Cooper RA, Udaltsova N, Armstrong MA (2003). The Early Repolarization Normal Variant Electrocardiogram: Correlates and Consequences. Am J Med.

[4] Haïssaguerre M, Derval N, Sacher F, Jesel L, Deisenhofer I, Roy L (2008). Sudden Cardiac Arrest associated with Early Repolarization. N Engl J Med.

[5] Diab OA, Allam RMA, Mohamed HG, Mohamed TR, Khalid SA (2015). Early Repolarization Pattern Is Associated with Increased Risk of Early Ventricular Arrhythmias during Acute ST Segment Elevation Myocardial Infarction. Ann Noninvasive Electrocardiol.

[6] Siebermair J, Sinner MF, Beckmann BM, Laubender RP, Martens E, Sattler S (2016). Early Repolarization Pattern is the Strongest Predictor of Arrhythmia Recurrence in Patients with Idiopathic Ventricular Fibrillation: Results from a Single Centre Long-Term Follow-Up Over 20 Years. Europace.

[7] Krahn AD, Healey JS, Chauhan V, Birnie DH, Simpson CS, Champagne J (2009). Systematic Assessment of Patients with Unexplained Cardiac Arrest: Cardiac Arrest Survivors With Preserved Ejection Fraction Registry (CASPER). Circulation.

[8] Tikkanen JT, Junttila MJ, Anttonen O, Aro AL, Luttinen S, Kerola T (2011). Early Repolarization: Electrocardiographic Phenotypes Associated with Favorable Long-Term Outcome. Circulation.

[9] Patton KK, Ellinor PT, Ezekowitz M, Kowey P, Lubitz SA, Perez M (2016). Electrocardiographic Early Repolarization: A Scientific Statement from the American Heart Association. Circulation.

[10] Haïssaguerre M, Sacher F, Nogami A, Komiya N, Bernard A, Probst V (2009). Characteristics of Recurrent Ventricular Fibrillation Associated with Inferolateral Early Repolarization Role of Drug Therapy. J Am Coll Cardiol.

[11] Liu LJ, Tang N, Bi WT, Zhang M, Deng XQ, Cheng YJ (2022). Association between Temporal Changes in Early Repolarization Pattern with Long-Term Cardiovascular Outcome: A Population-Based Cohort Study. J Am Heart Assoc.

[12] Rizzo C, Monitillo F, Iacoviello M (2016). 12-Lead Electrocardiogram Features of Arrhythmic Risk: A Focus on Early Repolarization. World J Cardiol.

[13] Lanza GA, Argirò A, Mollo R, De Vita A, Spera F, Golino M (2017). Six-Year Outcome of Subjects Without Overt Heart Disease With an Early Repolarization/J Wave Electrocardiographic Pattern. Am J Cardiol.

