# Supplementary material for: Mortalidade e Arritmia Ventricular em Pacientes com Repolarização Precoce
Source: Arq Bras Cardiol. 2025 Apr 17;122(4):e20240516. [Article in Portuguese] doi: 10.36660/abc.20240516 (PMC12108119; doi:10.36660/abc.20240516)
Supplement: Translation

# Translation: Mortality and Ventricular Arrhythmia in Patients with Early Ventricular Repolarization

**DOI:** 10.36660/abc.20240516i

**Authors:** Hugo Baldisserotto, Barbara Adelmann de Lima, Marco Aurélio Lumertz Saffi, Anderson Donelli da Silveira, Marcia Leonardi Baldisserotto, Tiago Luiz Luz Leiria

**Affiliations:** 1 Brazil Programa de Pós-Graduação em Ciências da Saúde: Cardiologia e Ciências Cardiovasculares, Universidade Federal do Rio Grande do Sul, Porto Alegre, RS – Brazil; 2 Brazil Pontifícia Universidade Católica do Rio Grande do Sul, Porto Alegre, RS – Brazil; 3 Brazil Departamento de Psicometria, Universidade Federal do Rio de Janeiro (UFRJ), Rio de Janeiro, RJ – Brazil

**Keywords:** Electrocardiography, Mortality, Cardiac Arrhythmias

## Abstract

**Background:**

Recent research has linked early repolarization (ER) with increased ventricular fibrillation risk, especially in leads V1-V3 and in inferior and lateral regions. However, data on the Brazilian population are limited.

**Objective:**

To estimate the impact of ER on survival and the occurrence of ventricular arrhythmias (VA) in patients over a 10-year period at a university hospital.

**Methods:**

This retrospective cohort study included patients with ER on electrocardiogram from the hospital database. Descriptive analysis was conducted to describe patients’ profile and characteristics. Kaplan-Meier method was utilized to analyze survival curves, with the log-rank test employed to assess differences between ER types. Cox regression models were applied to evaluate the risks of death and VA, calculating both gross and adjusted hazard ratios. The level of significance adopted in the statistical analysis was 5%.

**Results:**

The study population was predominantly male, average age of 45.6 years; 2.7% experienced VA (five in the group with lateral ER, four in the group with inferior ER group, and four in the ER inferolateral group). Significant differences were observed in age, sex, and HFrEF between the groups. Regarding all-cause mortality, 2.3% of patients died (five in the group with lateral ER, one in the group with inferior ER group, and five in the ER inferolateral group). Only age showed a statistically significant difference. There were significant differences in both death and VA between the groups (p=0.7 and p=0.5, respectively).

**Conclusion:**

ER did not lead to a higher incidence of VA or all-cause mortality in this cohort.

## Introduction

The prevalence of early repolarization (ER) ranges from 2.3% to 29.3%, varying with age, race, and sex.^1^ ER is also commonly found in high-performance athletes, with an incidence is around 1/3. However, the yearly incidence of sudden cardiac death in this population is very low.^2^ For many years, ER was considered a benign finding on the electrocardiogram (ECG), but recent studies suggest that this pattern in leads other than V1-V3 (especially inferior leads) is associated with a higher risk of ventricular arrhythmias and sudden death.^1,3-5^

Haïssaguerre et al.^4^ found a high prevalence of ER in patients who were resuscitated after cardiac arrest due to ventricular fibrillation (VF), and patients with ER presented twice the risk of VF recurrence.^4^ In the CASPER study, patients who had aborted sudden death with structurally normal hearts, ER was found as the cause in 7.9% of the sample.^6^ The degree of elevation of point J in the inferior leads can correlate with the magnitude of risk. The relative risk of death was 1.3 in those with 0.1 mV and three times in those with 0.2 mV;^1^ the risk of ventricular arrhythmia was bigger in those with 0.2 mV, with the inclination of the ST segment resulting in a meaningful prognosis, with a horizontal or downward orientation associated with a higher arrhythmic risk.^7^

Screening and management of patients with ER and the risk of sudden death are a challenge.^8^ The American Heart Association (AHA) recommends that, when detected, standard ER should be investigated in case of unexplained syncope or family history of sudden death and arrhythmogenic ECG features.^8^ The low incidence of arrhythmic events also presents a major challenge for effective interventions that can reduce arrhythmic risk.^2^

Although quinidine and isoproterenol can be effective in managing patients with VF, the data supporting their use remains limited to small samples.^9,10^ Due to the limited data in the literature, especially concerning the Brazilian population, this study aims to evaluate whether patients with different types of ER present a higher risk of ventricular arrhythmias and death.

## Methods

Temporal changes in the ER pattern were not assessed in this study; only a single ECG was evaluated. ER was classified based on the morphology observed in leads: lateral (leads I, aVL, V5-V6), inferior (leads II, III, aVF), and infero-lateral (combination of these leads).

This retrospective cohort study included 478 patients from a university hospital with an electrocardiographic diagnosis of early ventricular repolarization between 2011 and 2022. The study was conducted in accordance with regulatory guidelines involving human subjects, and the research project was approved by the Institutional Research Ethics Committee (number 2021/0585).

Inclusion criteria encompassed all patients diagnosed (by cardiologists) with early ventricular repolarization (J point elevation ≥ 1 mm in at least two contiguous leads, with a slurring or notching pattern, excluding leads V1 to V3 – Figure 1) who underwent ECG evaluation at the university hospital and were older than 18 years between 2011 and 2022. Exclusion criteria comprised the impossibility of performing an electrocardiogram for measures of ventricular repolarization parameters, secondary changes from ventricular repolarization, and patients with pacemakers and other cardiac devices.

**Figure 1:** – Electrocardiographic patterns of early repolarization.

The search for the term “early repolarization” through the hospital database retrieved 2,172 patients. Of those, 923 were selected for review based on the inclusion criteria. After thorough evaluation, 478 patients were diagnosed with ER.

### Variables of the study

Exposure variables included early ventricular repolarization, classified into three groups: lateral, inferior, and inferolateral. Outcome variables included sustained ventricular arrhythmia and all-cause mortality. Confounding variables controlled included age and sex.

### Data analysis

An exploratory analysis of sample characteristics was conducted, and the results were presented as proportions or averages, depending on the nature of the variable. Subsequently, the characteristics of the sample were stratified according to deaths and ventricular arrhythmias for further analysis. Group differences were assessed using the chi-square test for categorical data and the t-test for continuous data, with statistical significance set at p-value ≤ 0.05. A control population was used for comparative analysis.

For survival analysis concerning various types of ER, time was calculated in months (0-120 months), and the event (failure) was defined as patient death during the 10-year follow-up period. Patients who survived beyond the follow-up period were censored on the right. Similarly, for analysis of time to ventricular arrhythmia, time was calculated in months, and the event (failure) was defined as the occurrence of arrhythmia during the 10-year follow-up. Patients without ventricular arrhythmia after the follow-up period were considered censored data on the right.

To estimate survival probability and cumulative risk over time for death and ventricular arrhythmia in relation to the types of ER, the Kaplan-Meier method was employed. Differences between survival curves for both outcomes were assessed using the log-rank test, with significance set at p-value ≤ 0.05.

To analyze the relationship between ER and time until death or arrhythmia occurrence in the study population, Cox regression was utilized. Gross and adjusted hazard ratios (HR) were obtained for confounding variables, along with 95% confidence intervals (95 CI). Data analysis was performed using R version 4.3.0.

## Results

During follow-up, ventricular arrhythmias included sustained ventricular tachycardia and ventricular fibrillation as defined by ICD-10. Causes of death were primarily cardiac conditions, including sudden cardiac arrest.

A total of 2172 patients with a previous diagnosis of ER on the ECG were initially identified from the hospital database. Of these, 941 patients were selected for review based on specific inclusion criteria. After thorough evaluation by three cardiologists, 478 patients were confirmed to have a genuine diagnosis of ER and were included in the study. The remaining patients who did not fulfill the criteria for ER were used as controls (Figure 2 and Central Illustration).

**Figure 2:** – Flowchart of patient selection from the sample. ER: early repolarization; ECG: electrocardiogram.

**Central Illustration:** : Mortality and Ventricular Arrhythmia in Patients with Early Ventricular Repolarization

The study population was predominantly male (74%) with an average age of 45.6 years. Among them, 21.5% were active smokers, and 43% had some high school education. The prevalence of comorbidities such as diabetes mellitus, systemic arterial hypertension, dyslipidemia, coronary artery disease, and heart failure with reduced ejection fraction varied within the sample, with the lateral ER group showing a higher prevalence of systemic arterial hypertension (Table 1).

**Table 1:** – Characteristics of the study population

	Lateral	Inferior	Inferolateral	Total
**Variables**	(n = 170)	(n = 98)	(n = 210)	n = 478
**Age**	48.5±4.95	45.5±7.78	43.2±5.46	45.57±6.56
**Education**				
Some primary education	79 (46)	40 (41)	87 (41)	206
Primary education	36 (20.4)	19 (19)	45 (21)	100
Secondary education	29 (17)	19 (19)	38 (18)	86
Tertiary education	11 (6.4)	9 (9.1)	12 (5.7)	32
Unknown	7 (4.1)	9 (9.1)	17 (8.9)	33
**Sex**				
Male	130 (76)	70 (71)	154 (73)	354
Female	40 (24)	28 (29)	56 (27)	124
**Diabetes Mellitus**	35 (20)	16 (16)	42 (20)	93
**SAH**	86 (50)	32 (33)	75 (36)	193
**Dyslipidemia**	14 (8.2)	5 (5.1)	12 (5.7)	31
**Active smoker**	35 (20)	19 (19)	49 (23)	103
**CAD**	17 (10)	12 (12)	14 (6.7)	43
**HFrEF**	5 (3)	2 (2)	2 (1)	9

SAH: Systemic arterial hypertension; CAD: coronary artery disease; HFrEF: heart failure with reduced ejection fraction.

During the follow-up period, 13 patients (2.7%) experienced ventricular arrhythmias, with varying incidence between ER groups. Significant differences were observed in age, sex, and heart failure with reduced ejection fraction between the groups Regarding mortality, 11 patients (2.3%) died, with differences in age between the ER groups (Table 2).

**Table 2:** – Characteristics of patients (n=11) who died during the follow-up

	All-cause mortality		Ventricular arrhythmia	
	(n=11)	p-value^**c**^	(n=13) p-value^**d**^	
Age	59±10.24	0.0012^b^	35.3±17.50	0.0519^b^
**Education**		0.3140^a^		0.2813^a^
Some primary education	3 (27)		8 (62)	
Primary education	2 (18)		2 (15)	
Secondary education	6 (55)		3 (23)	
Tertiary education	0		1 (8)	
Unknown	0		0	
**Type of ER**		0.6201^a^		0.4758^a^
Lateral	5 (45)		5 (38)	
Inferior	1 (9)		4 (31)	
Inferolateral	5 (45)		4 (31)	
**Sex**		1^a^		0.0255^a^
Male	8 (73)		13 (100)	
Female	3 (27)		0	
**Diabetes mellitus**	2 (18)	1^a^	1 (8)	0.4786^a^
SAH*	5 (45)	0.9709^a^	6 (46)	0.7765^a^
**Dyslipidemia**	0	1^a^	1 (8)	0.5865^a^
**Active smoker**	3 (27)	0.8334^a^	2 (15)	0.1588^a^
**CAD**	0	0.6099^a^	3 (23)	0.1031^a^
**HFrEF**	1 (9)	0.4504^a^	2 (15)	0.0221^a^

*SAH: Systemic arterial hypertension; CAD: coronary artery disease; HFrEF: heart failure with reduced ejection fraction; ER: early repolarization; a: chi-square test; b: Student’s t-test; c: compared with the non-death group; d: compared with the group without arrhythmia ventricular.

The Kaplan-Meier survival analysis demonstrated no statistically significant differences in survival rates between the groups of patients with different types of ER and the control group (p-value = 0.7 and p-value = 0.5 for mortality and ventricular arrhythmia, respectively). However, the analysis revealed varying cumulative risk for death and ventricular arrhythmia between different ER groups (Figure 3).

**Figure 3:** – Kaplan-Meier survival. ER: early repolarization.

Cox regression analysis showed no statistically significant hazard ratios for different types of ER regarding mortality or ventricular arrhythmias. Only age demonstrated a significant hazard ratio for both outcomes (Table 3).

**Table 3:** – Crude and adjusted models (Cox regression) from the relation between early repolarization and death and ventricular arrhythmia for study population

	Death		Arrhythmia	
	HR Gross	HR Adjusted	HR Gross	HR Adjusted
**Early repolarization**				
Lateral	HR= 1.000	HR=1.000	HR=1.000	HR= 1.000
Inferior	HR=0.419 (IC= 0.049 - 3.589)	HR= 0.528 (IC= 0.061 - 4.580)	HR= 1.537 (IC= 0.412 - 5.731)	HR= 1.278 (IC= 0.336 - 4.856)
Inferolateral	HR=0.928 (IC= 0.268 - 3.211)	HR= 1.242 (IC= 0.354 - 4.365)	HR= 0.670 (IC= 0.180 - 2.497)	HR= 0.543 (IC= 0.143 - 2.054)
**Age**		HR= 1.080 (IC= 1.027- 1.135)	HR= 0.959 (IC= 0.925 - 0.995)	
**Sex**				
Male		HR=1.000		
Female		HR= 0.988 (IC= 0.2605 - 3.750)		

## Discussion

Recent findings from Liu et al.^11^ suggest that ER is a transient phenomenon, appearing in baseline ECGs but potentially resolving in subsequent measurements. This transient nature could indicate a benign course, as discussed in their cohort study.

The elevation of the J point in the ECG in 12 leads, compatible with ER, has traditionally been viewed as an innocent finding in healthy individuals.^12,13^

The study sample, as expected, consisted of middle-aged individuals with fewer comorbidities than typically seen in cardiology studies. Although not a randomized study, the characteristics of the groups were very similar, with only the lateral ER group showing a higher incidence of hypertensive patients, reducing the effect of selection bias, although it still exists. Previous studies have reported average ages of ER populations equal to or very similar to the sample in this study.^1^

When analyzing the characteristics of the sample that died, age resulted in a statistically significant difference, confirming the expected association between older age and higher mortality risk. Regarding the incidence of ventricular arrhythmias, sex and heart failure with reduced ejection fraction (HFrEF) resulted in statistically significant differences. Patients with ventricular arrhythmia consisted of young males, with a notable percentage presenting HFrEF, indicating a predisposition to ventricular arrhythmogenesis through various cardiomyopathy mechanisms. Age, after adjustment in the Cox model, emerged as a protective factor against ventricular arrhythmia.^3^

Survival curves analyzed using the Kaplan-Meier method revealed high survival rates across all groups, with no statistically significant differences (p=0.7). However, the group with inferolateral ER exhibited the highest cumulative risk. Consistent with the findings from Tikkanen et al.^1^ in 2009, patients with inferior ER showed a higher rate of cardiovascular mortality, especially when the J point was higher than 2mm. However, when analyzing mortality from all causes, no significant difference was found between the groups with lateral ER and inferior ER. The percentage of the sample free of ventricular arrhythmia was high across all groups, with no statistically significant differences (p=0.5). Unlike mortality, patients with inferior ER exhibited a greater cumulative risk of ventricular arrhythmia, while the infero-lateral group showed the lowest risk. Similar findings were reported by Tikkanen et al.^1^ in 2009 regarding ventricular arrhythmia, with a greater incidence observed in the inferior ER group when the J point was higher than 2mm.^1^

Although mortality did not significantly differ between groups, it was interesting to note the difference observed in the HR adjusted for age and sex in the group with inferolateral ER. This finding suggests a potential risk factor with 1.2 times more chance of death. However, the wide confidence interval, including zero, may be attributed to the small number of deaths. More recently, Haïssaguerre et al.^4^ observed a higher frequency of inferolateral ER in patients with sudden death aborted by ventricular tachycardia, corroborating the findings of this study that ER category is associated with a greater risk of ventricular arrhythmia and death.^4^

Despite making important contributions to this field of knowledge, this study has several limitations. Data were obtained retrospectively through the analysis of medical records, and many details depended on third-party descriptions, which sometimes resulted in incomplete or missing information. Data extraction from medical records was also limited by technical issues and restricted access to these documents. Additionally, the use of secondary data, such as the SIM, due to scarce information regarding deaths made it challenging to categorize the cause of death. Another limitation was the low number of outcomes (death and ventricular arrhythmia), which could compromise the sample’s power to detect statistically significant differences. It is essential for clinicians, particularly cardiologists, to recognize the significance of this new evidence and pay close attention to patients with these electrocardiographic findings.

Also, the design of a retrospective cohort study has several limitations that warrant consideration. Firstly, its retrospective design relying on pre-existing data from medical records introduces potential biases and limitations in data completeness and accuracy. The relatively small sample size and limited number of outcomes, particularly deaths and ventricular arrhythmias, may have impacted the study’s statistical power and ability to detect significant differences between ER groups. Additionally, the study’s single-center nature and exclusion criteria may limit the generalizability of findings to broader populations. Data quality and availability, and potential confounding factors not fully controlled, further add complexity to the interpretation of results. The observational nature of the study precludes the establishment of causal relationships between ER and outcomes. Longer follow-up periods and prospective, multicenter studies are needed to validate findings and address these limitations comprehensively.

## Conclusion

In this study, we observed that patients with early ventricular repolarization, particularly in the lateral and inferior leads, did not exhibit a significantly higher incidence of ventricular arrhythmias or mortality. These findings suggest that the presence of ER alone may not be a strong predictor of adverse outcomes in our study population. However, further prospective and randomized studies are warranted to validate these results and inform clinical practice regarding the management of patients with early ventricular repolarization.
